# Metabolomics as an Important Tool for Determining the Mechanisms of Human Skeletal Muscle Deconditioning

**DOI:** 10.3390/ijms222413575

**Published:** 2021-12-17

**Authors:** Isabelle Alldritt, Paul L. Greenhaff, Daniel J. Wilkinson

**Affiliations:** 1 MRC Versus Arthritis Centre for Musculoskeletal Ageing Research, University of Nottingham, Nottingham NG7 2UH, UK; isabelle.alldritt@nottingham.ac.uk (I.A.); paul.greenhaff@nottingham.ac.uk (P.L.G.); 2 NIHR Nottingham Biomedical Research Centre, University of Nottingham, Nottingham NG7 2UH, UK; 3 School of Medicine, Royal Derby Hospital Centre, University of Nottingham, Derby DE22 3SY, UK; 4 School of Life Sciences, Queen’s Medical Centre, University of Nottingham, Nottingham NG7 2UH, UK

**Keywords:** metabolomics, skeletal muscle, sarcopenia, cachexia, inactivity, mass spectrometry

## Abstract

Muscle deconditioning impairs both locomotor function and metabolic health, and is associated with reduced quality life and increased mortality rates. Despite an appreciation of the existence of phenomena such as muscle anabolic resistance, mitophagy, and insulin resistance with age and disease in humans, little is known about the mechanisms responsible for these negative traits. With the complexities surrounding these unknowns and the lack of progress to date in development of effective interventions, there is a need for alternative approaches. Metabolomics is the study of the full array of metabolites within cells or tissues, which collectively constitute the metabolome. As metabolomics allows for the assessment of the cellular metabolic state in response to physiological stimuli, any chronic change in the metabolome is likely to reflect adaptation in the physiological phenotype of an organism. This, therefore, provides a holistic and unbiased approach that could be applied to potentially uncover important novel facets in the pathophysiology of muscle decline in ageing and disease, as well as identifying prognostic markers of those at risk of decline. This review will aim to highlight the current knowledge and potential impact of metabolomics in the study of muscle mass loss and deconditioning in humans and will highlight key areas for future research.

## 1. Introduction

Skeletal muscle accounts for approximately 45% of total body mass and contains 50–75% of all proteins as well as a significant proportion of the body store of glycogen and lipid. Skeletal muscle not only enables body movement, maintenance of posture, and mechanical ventilation of the lungs, it is also central in maintaining numerous metabolic functions including energy homeostasis, insulin sensitivity, amino acid metabolism, and inter-organ communication [1,2]. Furthermore, it acts as a reserve for glucose and amino acids for other tissues and organs during periods of illness or trauma [2,3], and provides compensatory metabolic function to failing organs [4]. In certain circumstances, including ageing [5], acute trauma and inflammation, chronic disease [6,7,8,9], or following prolonged periods of disuse, such as bedrest, immobilisation or spaceflight [10], it is common to experience muscle level deconditioning and a physical decline in locomotor function. The characteristics of deconditioning are widespread, including reduced cardiorespiratory fitness and increased systemic insulin resistance [11]. Notably, deconditioning of muscle is also associated with the loss of mass and strength and increased weakness and fatigue during sustained movement, thereby reducing quality of life [12]. Such negative changes increase the risk of falls and subsequent disability, and are associated with increased prevalence of comorbidities such as diabetes or cardiovascular diseases [13,14], hospitalisation, and increased mortality rates [15]. Ordinarily, in grown adults, skeletal muscle mass is maintained by an exquisite balance between rates of muscle protein synthesis (MPS) and muscle protein breakdown (MPB), such that the net protein balance remains zero across the day [16]. However, in deconditioned muscle the balance between MPS and MPB is disrupted, leading to a net negative protein balance and the subsequent loss of muscle mass (see the following reviews for details [17,18]).

Sarcopenia is the progressive loss of muscle mass and strength with age, which is accelerated in cachexia and wasting conditions associated with chronic disease. Whilst several consensus definitions of sarcopenia have been published [5,19,20,21,22,23], the prevalence of sarcopenia or cachexia within study cohorts varies wildly based on the definition used [24,25,26]. Indeed, there is little agreement in the identification of wasting by consensus definition versus clinical assessment [27], drawing their clinical application into debate. There is a clear need for improved diagnostic criteria but the search for objective biomarkers of muscle atrophy has been inconclusive [28,29,30,31,32,33,34,35,36]. Recently, some metabolic processes likely to be dysregulated in the development of these conditions have been identified including protein turnover and energy/fuel metabolism [37]. However, despite these advancements, the importance and contribution of these mechanisms in the aetiology of deconditioning is still unknown and as a result have so far failed to identify effective interventions for treating or reversing wasting (e.g., the failure of recent clinical trials [38,39]). For example, the best approach to combat (yet not overcome) declines in muscle mass remains the maintenance of adequate nutritional intake and physical activity [40,41]. Additionally, it is unknown whether all human wasting conditions, due to healthy ageing, disuse or disease, are regulated via distinct mechanisms, or whether there are aspects of their pathology that may overlap. Given the complexities of these unknowns, an alternative approach allowing for a holistic and unbiased view is clearly needed.

## 2. The Applications of Metabolomics

OMICs refers to technologies aimed at the universal detection of molecules within a biological sample [42]. They are often high-throughput and generate very large datasets that provide biological insights and associations based on statistical inference [43]. Rather than beginning from a pre-determined hypothesis, OMICs experiments instead focus on understanding a complex system by looking at it as a whole and exploring the roles and relationships between molecules within that system, using the acquired data to define a hypothesis that can be tested further [44]. Of the numerous OMICs techniques available [45], those most routinely utilised in the literature are genomics, transcriptomics, proteomics, and metabolomics.

While genomics, proteomics, and transcriptomics are powerful techniques that have enabled significant advances in the field of biomedical sciences, such as providing diagnostic protein signatures in bladder [46], ovarian [47] and lung cancers [48], or transcriptomic signatures of ageing systems, health and longevity [49,50,51]. The data gathered from such experiments only represents alterations that predispose an organism’s phenotype, and so findings do not always correlate directly with the phenotypic changes observed within conditions [45]. Metabolomics, in contrast, is a comparatively new field, first described in 1998 [52], which studies the metabolome, the full complement of small molecules present in a cell that participate in metabolic reactions and that are required for the normal maintenance, growth, and function of the cell [53]. Metabolites represent the end points of cellular regulatory networks [54], and perturbations to the genome, transcriptome, or proteome are represented in the downstream metabolome, making it the potentially crucial linkage between environment, genotype, and phenotype [55].

Untargeted metabolomics analyses the full set of measurable metabolic features in a sample, including uncharacterised metabolites, in an unbiased way allowing for the identification of relative changes in complex samples [56]. These changes can be used to infer the mechanism of disease progression and identify possible therapeutic targets. The use of untargeted metabolomics in the clinical study of complex diseases has been growing over the past 20 years [57]. For example, previous work has been able to identify biomarkers indicative of early-stage Parkinson’s disease [58], a panel of ten lipids capable of predicting the presence of preclinical Alzheimer’s disease with a high degree of accuracy [59], novel metabolic disturbances associated with amyotrophic lateral sclerosis [60], and a panel of amino acid and pentose phosphate metabolite biomarkers that are associated with the onset of non-alcoholic fatty liver disease [61]. A key component of this process is the ability of untargeted metabolomics to identify unknown compounds. This may be particularly important in deconditioning, as although the involvement of metabolic dysregulation is clear, no successful interventions or treatments have been developed thus far. It is possible that a key insight is yet to be uncovered by using a novel approach.

In contrast, targeted metabolomics provides a quantitative measure of defined groups of metabolites [62]. By its nature, targeted metabolomics require prior knowledge of metabolites and limit the coverage of the metabolome, which in turn limits the discovery of novel metabolic responses. However, it may reveal new associations between the defined metabolites and make the identification of valid biomarkers easier [63]. As such, targeted analysis is well suited to hypothesis testing studies and can be used to validate the results of previous untargeted work.

Although there remain limitations to metabolomics, particularly associated with the annotation and coverage of the human metabolome (when compared to the proteome and transcriptome, for example) [64], it has been gaining interest over the years due to its ability to add insight into disease aetiology and identify metabolic signatures that can function as prognostic or diagnostic tools. The following will highlight current progress of metabolomics research within the field of muscle deconditioning.

## 3. Metabolomics as a Tool to Study Muscle Level Deconditioning

### 3.1. Dysregulated Amino Acid Metabolism

Several metabolomics studies have highlighted abnormal amino acid level in deconditioned muscle. For example, circulating plasma concentrations of seven branched chain amino acids (BCAAs) or BCAA-related metabolites were found to be significantly associated with both muscle cross-sectional area (CSA) and fat free mass index in functionally limited older adults, indicating a role for these metabolites in sarcopenia [65]. Of these, four compounds (leucine and its metabolite α-hydroxyisocaproate, and two tryptophan-related metabolites, C-glycosyltryptophan, and indolepropionate) accounted for 21% of variability in muscle CSA, suggesting these metabolites in particular could be important in classifying changes in CSA. BCAAs were also among the 10 metabolites found to be most associated with muscle mass in middle aged women [66]. These studies do not link amino acids to muscle function, a key component of sarcopenia; however, when muscle quality, determined by the ratio of quadriceps strength to thigh CSA, was assessed, leucine, isoleucine, and tryptophan were all found to be present at significantly higher plasma concentrations in participants with low muscle quality than their age-matched controls [67]. Targeted studies support these findings: low concentrations of serum BCAA and EAA have been linked to both lower skeletal muscle index and functional ability in older adults [68]; and fasting BCAA plasma concentrations were significantly lower in sarcopenic women with poor physical performance than sarcopenic women with higher performance metrics or non-sarcopenic women [69]. Although it should be noted that participants’ daily protein intake was not recorded, and these levels may simply reflect differences in habitual protein consumption or habitual energy balance. Non-fasting BCAA plasma concentrations were also significantly lower in sarcopenic than non-sarcopenic individuals; however, no specific data were collected regarding the last meal eaten before the blood sample was taken. In addition, a general dietary recall found absolute protein intake was significantly lower in sarcopenic participants. Therefore, lower amino acids could simply be reflective of the reduced protein content of the last meal. The authors argue that non-fasting concentrations reflect the postprandial response to protein intake, but this could be better assessed by using a standardised and controlled diet.

Other non-BCAA or EAA related metabolites have also been linked to muscle deconditioning with age. One study identified proline as an independent risk factor sarcopenia [70], while another identified nine metabolites significantly associated with both muscle mass and function, including aspartic acid and glutamate, which were both negatively associated with grip strength and appendicular lean mass albeit the study only recruited young women [71]. Eight pathways were significantly associated with the loss of muscle mass and function, of which seven involved glutamic acid and/or aspartic acid. Of these pathways, the majority related to amino acid metabolism, supporting a role for aberrant amino acid metabolism in sarcopenia [71]. While these disturbances were identified only in young women, targeted analysis later revealed significantly increased serum concentrations of glutamic acid, asparagine, and aspartic acid in elderly individuals with sarcopenia [72]. Moreover, in severely frail elderly patients, significant differences in the plasma concentration of 11 amino acids was also identified [73]. EAAs were present at a significantly lower concentration in the plasma, with a 20.2% reduction in total EAA level in the frail group compared to a non-frail control. In addition, EAA, BCAA, and tryptophan levels were strongly correlated with BMI in the frail group. Although this study focused on frailty, which is distinct from sarcopenia, it further supports that amino acids provide a signature for low physical function and suggests that dysregulated amino acid metabolism may be common across conditions associated with deconditioning.

In line with these findings in sarcopenia and age-related muscle loss, dysregulated amino acid metabolism has also been noted in cachexia. For example, in cancer patients the most prominent metabolic alteration associated with cachexia was found to be a decrease in the plasma concentration of amino acids, particularly arginine, tryptophan, and threonine, which had a 0.4-fold reduction compared to non-cachexic cancer controls [74]. Urinary metabolomics was able to effectively discriminate between cachectic and non-cachectic groups, and metabolites that differed between groups were largely involved in ketone body and amino acid metabolism pathways [75]. However, these changes may not be specific to skeletal muscle, and the differences in metabolite concentration did not remain significant after adjusting for multiple testing, such that further validation of these findings is required. Finally, Yang et al. identified 15 metabolites to be distinct biomarkers for cancer cachexia, including increases in the levels of lysine, isoleucine, and tyrosine and decreases in leucine [76]. The metabolic pathways most disturbed in cachexia included the metabolism of several amino acids, and the synthesis and degradation of BCAAs.

Likewise, amino acid metabolism is clearly affected by daily energy expenditure. One study noted an inverse association between BCAA concentration and physical activity alongside lower plasma concentrations of several intermediates of BCAA metabolism in more active subjects [77], while another found BCAA plasma concentration decreased in highly active individuals compared to their sedentary counterparts [78], which suggests BCAA plasma concentration is linked in some way to muscle function. Additionally, in older adults, two weeks of deconditioning resulted in a significant increase in plasma glutamine (1.3-fold) and methionine (1.2-fold), which did not return to baseline values following a recovery period [79]. Proline has also been highlighted as a marker for both physical inactivity and cachexia. Reductions in plasma proline concentration were associated with higher physical activity and shorter sitting times [78], and significant increases in proline concentrations were observed in COPD patients with cachexia [80]. Proline is a glycogenic amino acid, providing a possible link between dysregulated skeletal muscle metabolism and glucose homeostasis in wasting.

While metabolomics studies investigating the effects of mechanical unloading in humans are lacking, a key limitation in this area, these results from healthy ageing and cachexia provide preliminary evidence that amino acid metabolism is dysregulated in skeletal muscle following periods of disuse, and perhaps a link in terms of metabolic disturbances within the regulation of amino acid metabolism. These observations of dysregulated amino acid metabolism could be related to a number of known metabolic disturbances present in muscle wasting conditions or could be indicative of an as yet unknown mechanism common across wasting conditions.

#### Biological Links to Explain Metabolomic Outcomes

Firstly, the development of anabolic resistance, the blunted response of muscle to nutritive or contractile stimuli, is a hallmark of many muscle wasting states [81]. For example, the response of skeletal muscle to nutrition has been shown to be significantly blunted in older adults receiving comparable absolute doses of protein or AAs [82,83,84,85,86], and the protein intake required to achieve maximal postprandial rates of MPS in skeletal muscle is 68 and 140% greater relative to body mass and lean body mass, respectively, in old compared to young men [84]. Moreover, in pancreatic and colorectal cancer patients, cachectic individuals were found to have higher basal whole-body protein turnover rates than age matched control groups, and protein synthesis did not respond to sip feeding in either patient group [87,88]. Given that tumour resection in colorectal cancer patients restored postprandial MPS and reduced MPB such that rates were comparable with the control group in both the fasted and fed states, anabolic resistance seems likely to be key in the development of cachexia. Likewise, a 5-day period of immobilisation was sufficient to reduce postprandial MPS rates by 53% in healthy young men [89] and even a reduction in daily step count for 2 weeks induced a 26% decline in postprandial MPS rates [90]. The rapid development of anabolic resistance in disuse associated wasting points to its importance in the aetiology of deconditioning. BCAAs, particularly leucine, are instrumental in driving MPS [91] and increase following MPB proportional to fat free mass [92]. The observed signatures of altered AA metabolism may therefore be indicative of a lack of incorporation after feeding leading to low MPS rates, or of increased MPB rates in muscle.

Alternatively, the increased presence of BCAAs may promote insulin resistance through increased mTOR activation [93]. A blunted response to the inhibitive effect of insulin on MPB has been observed in older adults; compared to basal rates, administration of 15¼ IU/mL insulin lowered protein breakdown rates by 47% in young men, but only by 12% in old men [94]. Similarly, whole body and peripheral insulin resistance have been noted after acute and chronic periods of step count reduction [95] and immobilisation [96], which develops rapidly [97]. The cellular and molecular mechanistic basis of these pathophysiological events are unclear.

Abnormal glutamic acid metabolism and associated AA’s may be reflective of depressed energy metabolism pathways in muscle, as skeletal muscle acts as a major sink for glutamic acid where it plays a central role in energy provision [98]. Glutamine is a regulator of MPS and MPB; therefore, increased plasma glutamine following deconditioning may reflect low stores of in skeletal muscle, and the subsequent dysregulation of protein turnover providing a mechanism driving early muscle loss [99]. However, there are limitations to the conclusions that can be drawn brought about by study design. The cross-sectional nature of many studies means that although amino acids may provide a metabolic signature for a range of wasting conditions, causal links between abnormal amino acid metabolism and muscle wasting cannot be established and the precise role of amino acids in the wasting process remains unclear.

### 3.2. Lipid Metabolism

One of the advantages of an untargeted approach is its unbiased nature, allowing a range of metabolite classes to be identified from a single sample. This provides an opportunity to investigate wider metabolic derangements in wasting conditions. Increased intramyocellular lipid (IMCL) content is a common feature in deconditioning [100,101,102]. It is particularly prevalent in ageing; compared to young individuals, older adults display a significant increase in the size and density but not quantity of IMCLs [103], and both older men and women have a higher percentage non-contractile area (15.6% and 13.0%, respectively) in skeletal muscle than their young counterparts (6.0%) [104]. Similarly, IMCL level was found to be approximately 35% greater in cancer patients with cachexia than without [105], and a progressive rise in the number of lipid droplets in skeletal muscle with the progression of cachexia has been noted; although, droplet diameter did not increase with disease progression as it does in sarcopenia [106].

Whether the increased presence of IMCL in ageing and disease is a driver of pathophysiology or simply a consequence of changes in habitual energy intake and physical activity levels is currently unknown. Nevertheless, in line with the above, a recent metabolomics study showed total abundance of major phospholipid classes in skeletal muscle was increased in sarcopenic elderly when compared to their healthy counterparts [107]. Total phosphatidylcholine and phosphatidylethanolamine levels were negatively associated with muscle volume and peak power, suggesting they may be related to impaired muscle function and loss of muscle mass, however validation in a larger cohort is required to confirm the metabolic signature. Forty metabolites were identified as being strongly associated with cancer cachexia, most of which were classed as lipids or fatty acids, and six metabolites, including two phospholipids and two fatty acids, formed a distinct metabolic signature for cachexia [108]. Two of the most abundant compounds in both the cachexia and non-cachexia groups, lysophosphatidylcholine (LPC) 16:0 and LPC 18:2, increased 1.34-fold and 1.75-fold with cachexia, respectively, indicating a significant shift in lipid metabolism. LPC have been implicated in inflammation, facilitating the release of proinflammatory cytokines [109]. Given that one important feature of cachexia is chronic systemic inflammation [110], increased LPC may be a key factor in its development. If validated in a larger cohort of patients, this signature may identify those at risk of developing cachexia; however, it should be noted that the metabolites may be solely indicative of adipose tissue wasting and not muscle loss, as no assessment of skeletal muscle was performed. Loss of white adipose tissue before reductions in skeletal muscle content have previously been observed in cachexia [111]. Additionally, increased IMCL content was linked to increasing lipolysis in other compartments of the body in cachexia patients [106]. In cancer patients with cachexia mRNA expression of hormone sensitive lipase (HSL), a lipase protein present in adipose tissue, was approximately 50% higher than non-cachectic counterparts and HSL protein expressed increased 2–2.5-fold between cachectic and non-cachectic cancer groups [112]. Similar increases were seen between patients with cachexia and cancer patients who had lost weight due to other factors, such as malnutrition, indicating a unique role for lipolysis in cachexia-associated weight loss [113]. Loss of adipose tissue therefore appears to be a critical component in early-stage cachexia and such metabolites may provide evidence of a unique aetiology, perhaps distinguishing it from other diseases associated with deconditioning.

Changes in lipid metabolism after periods of inactivity are less clear. While some studies show increased IMCL content, similar to in sarcopenia, following short- and long-term disuse [102,114,115], this may simply reflect excess energy balance. When individuals are maintained in energy balance during bedrest, IMCL does not accumulate despite a 29% decrease in whole-body insulin sensitivity and a decline in muscle oxidative capacity [116].

Although lipid metabolism is clearly changing, there are relatively few metabolomics studies focused on lipid metabolism and deconditioning. Thus far, metabolomics has identified common lipid signatures in deconditioning with a particular focus on phospholipids; however, more research is needed in this area to validate this signature and to provide mechanistic insight into the aetiology of deconditioning. Furthermore, metabolomics studies specifically linking lipid metabolism and decreased energy expenditure are lacking, a key area for improvement.

#### Biological Relevance of Dysfunctional Lipid Metabolism

The precise role of IMCL content in deconditioning is unclear. Some have linked excess IMCL content to increased insulin resistance [117,118] but how IMCL might drive this change is not yet known. Initially, increased lipid accumulation was suggested to disrupt whole-body glucose homeostasis and directly impact insulin sensitivity [119]. However, recently there is more evidence to suggest that the coating of perilipins on the surface of the droplets influences droplet characteristics, including inhibiting lipolysis, morphological changes including size and shape, and affects the droplets’ dynamic nature, thereby modulating insulin sensitivity in skeletal muscle [120].

This theory aligns with both changes in the size and shape of lipid droplets in poor quality muscle [103,106] and the so-called athletes’ paradox, the increase in IMCL content in insulin sensitive endurance trained athletes. Insulin sensitive, trained individuals and insulin resistant, non-trained individuals have similar muscle fat content, but in trained muscle, lipids are stored in small lipid droplets while in the untrained muscle lipids accumulate in large droplets [121]. Additionally, high levels of the perilipin PLIN5 and low levels of PLIN2 in the trained state, point to high promotion of lipid turnover, which is lacking in the untrained state [122]. Endurance training of insulin resistant individuals can shift lipid droplet storage towards an athlete-like pattern [123], further supporting a role for lipid metabolism in deconditioning.

However, the potential role of IMCL in the development of insulin resistance is complicated by the lack of IMCL accumulation when energy balance is maintained in disuse despite the rapid development of insulin resistance [116]. Increased IMCL content is therefore unlikely to be the primary cause of insulin resistance following disuse and instead may simply be a side of effect of maintaining a positive energy balance.

Under fasting conditions in healthy individuals, skeletal muscle relies on lipid oxidation for energy production [124,125]. An alternative explanation for the shift in lipid metabolism observed in deconditioning may therefore be related to a change in lipid utilisation in the fasted state.

### 3.3. Dysregulated Energy Metabolism

Deconditioning negatively impacts on mitochondrial mass and function. This dysregulation may lead to changes in lipid abundance and metabolism as discussed above; however, such losses can also occur independent of IMCL content [126]. Regardless of cause, a significant decline in mitochondrial content [103] and decreased mitochondrial function with increasing age [127], point to mitochondrial decay as a driving factor in sarcopenia. A significantly lower phosphocreatine recovery rate in pre-frail elderly at risk of sarcopenia than their active counterparts (40.82 s vs. 29.53 s, respectively) indicates a lower rate of muscle mitochondrial ATP production and/or a decrease in mitochondrial content [128]. Taken alongside the association of lower mitochondrial efficiency and capacity with slower walking speeds reported in older adults [129], this suggests reduced mitochondrial function and/or capacity contributes to the sarcopenic phenotype.

A similar phenomenon is seen following immobilisation. Citrate synthase activity decreased significantly and β-hydroxyacyl-coA dehydrogenase activity tended to decrease following 7 days of bedrest, indicative of a decline in mitochondrial mass [116]. Ten days of bedrest resulted in a significant reduction in mitochondrial content and respiration, normalised to content [130]. Fourteen days of bedrest was also characterised by a decrease in several variables associated with mitochondrial oxidative metabolism and mitochondrial biogenesis [131]. Finally, although substrate utilisation did not differ during exercise following unilateral leg immobilisation, plasma content of proteins related to energy metabolism was lower in immobilised than contralateral legs in both young and older men.

Less research has focused on the potential role of disrupted energy metabolism in cachexia. Some animal models have pointed to a role for mitochondrial degradation in cachexia without IMCL accumulation. For example, ATP synthesis rate was 47% lower in cachectic mice than controls and in addition genes related to mitochondrial function were altered in cachexia, with significant downregulation of genes involved in biogenesis and upregulation of genes related to uncoupling [132]. In human cachexia, abnormal mitochondrial structure and increased myofibrillar mitochondrial area in cachectic cancer patients when compared to weight stable controls were observed, supporting a role for mitochondrial dysfunction [133]. As mitochondrial degeneration precedes muscle atrophy [134], it could be a driving mechanism of cachexia. However, further research is required to definitively link changes in fuel metabolism with cachexia.

Mitochondrial dysregulation is reflected in the plasma metabolome. For example, in a study investigating metabolomic changes following 7 days of post-surgery bedrest, 557 metabolites changed significantly. Notably, short-chain acylcarnitines and derivatives of fatty acid dicarboxylates were elevated immediately following surgery and circulating expression remained high after bedrest [135]. Acylcarnitine is involved in the transport of fatty acids into the mitochondria for oxidation, so increased circulating levels of acylcarnitine have previously been suggested as a marker of mitochondrial dysfunction [136]. The observed increase in this study may reflect the importance of mitochondria in deconditioning. Supporting this, short-chain dicarboxylic and hydroxylated acylcarnitines were inversely associated with a decline in hand grip strength in men over 18 months and accounted for 16% of total variability in hand grip strength [137]. Given the similarities in metabolomics profiles between studies, dysfunctional mitochondrial bioenergetics may therefore be a key component of muscle level deconditioning across several conditions.

A comprehensive profiling of frail elderly versus healthy elderly and healthy young individuals found clear differences in the muscle metabolome, including lower levels of metabolites involved in the TCA cycle in older participants, which suggest impaired mitochondria or lower mitochondrial content with age [138]. Importantly, acylcarnitine level decreased significantly after resistance exercise training in both frail and healthy elderly, further supporting a role for dysfunctional mitochondrial bioenergetics in deconditioned muscle. However, it should be noted that as body composition was not accounted for in these measurements, this may simply be a consequence of muscle mass loss rather than a causative factor.

Metabolomics studies investigating changes to energy metabolism in sarcopenia and cachexia are lacking, but several studies do show positive changes relating to energy metabolism in sedentary individuals following increased physical activity. For example, urinary metabolomics profiling revealed short term intensive exercise alters mitochondrial bioenergetics, particularly those relating to glycolytic systems [139]. Likewise, sprint training induces changes in the urinary metabolome relating to bioenergetic pathways including TCA cycle intermediates and products of ATP degradation [140]. In particular, 2-oxoglutarate, a component of the TCA cycle, was elevated following physical activity alongside increases in metabolites released during its synthesis. Similar metabolic alterations are seen in the plasma metabolome [63]. Exercise induced the rapid upregulation of metabolic pathways responsible for substrate utilisation and increases in intermediate metabolites from adenine nucleotide catabolism and the TCA cycle. However, these changes only reflect acute metabolic adaptations to exercise. It is unclear whether similar positive changes are reflected in chronic metabolic responses, and therefore how important these metabolites are in the aetiology of deconditioning is yet to be determined.

### 3.4. Wider Metabolic Changes and the Decline in Neuromuscular Physiology

Metabolic dysfunction in muscle deconditioning extends past dysregulation of amino acids and lipids. For example, several aspects of the neuromuscular system are suggested to contribute to the loss of muscle strength in sarcopenia. Degradation of motor neurons and the subsequent denervation of muscle fibres may account for loss of muscle strength associated with increasing age, such as the decrease in doublet discharges from ∼46% of motor units in younger individuals to ∼25% in older individuals [141], or the decreased maximal firing rate and decline in maximal voluntary contraction observed with increasing age [142]. Denervation may be compensated for by the branching of surviving motor neurons resulting in increased motor unit size, as seen in healthy ageing where motor unit potential in non- and pre-sarcopenic men was larger than young men by 26 and 41%, respectively [143]. However, in sarcopenic men the motor unit potential was significantly smaller than the pre-sarcopenic group, suggesting failure to expand motor units and reinnervate muscle fibres plays a role in sarcopenic muscle and could provide a distinction between healthy ageing and the development of sarcopenia. Neuromuscular instability has also been associated with unloading with both 3 and 14 days of bedrest resulting in significant increases in the number of NCAM positive fibres [144,145]. Additionally, markers of denervation were observed in individuals with an inactive lifestyle, with a significantly higher percentage of denervated muscle fibres in sedentary seniors than both young and active older individuals [146]. In contrast, denervation does not appear to be a driver of cachexia. A recent study found that despite muscle fibre diameter being reduced by nearly 15% in cachectic patients, the morphology of neuromuscular junctions remained conserved across cachectic and non-cachectic individuals, with no evidence of pathology or denervation [147]. As such, investigating changes to the neuromuscular system may provide a distinction between cachexia and other muscle wasting conditions that represent unique aetiologies.

However, metabolomics studies investigating such disturbances are lacking. Murine models have been used to show the potential of untargeted analysis in this area, such as the identification of a 1.8-fold increase in acetylcholine in aged mice, likely representing a compensatory mechanism for the degeneration of neuromuscular junctions [148], or large-scale changes to cell metabolism identified in models of cachexia [149] or unloading [150], but whether these findings translate to human cohorts is unknown. Metabolic disturbances contribute significantly to muscle loss, therefore validating these signatures is essential in attempting to understand the mechanisms that drive changes in muscle level deconditioning.

## 4. Conclusions and Future Needs

Deconditioning of skeletal muscle is a key feature of chronic illness and physical inactivity and is accompanied by a loss in both muscle mass and physical function. Several metabolic phenomena have been proposed as contributing factors to muscle level deconditioning; however, the precise aetiology remains unclear. Given its complexity, a new approach is needed to fully understand these mechanisms and allow for the development of successful therapeutics.

**Figure 1:**
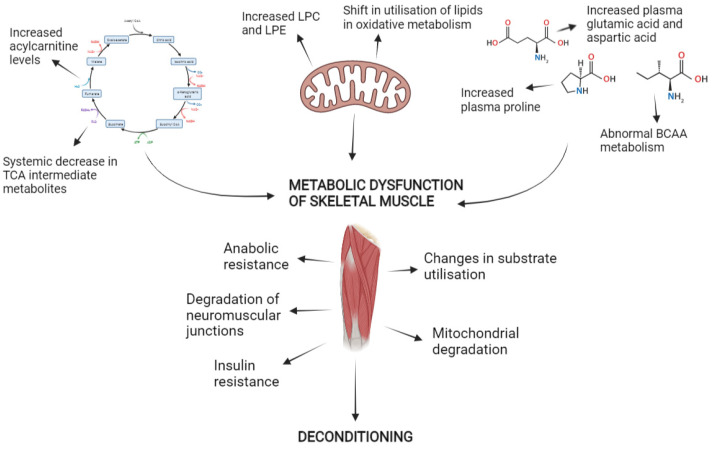
Summary of major findings from untargeted metabolomics work in the development of deconditioning.

Untargeted metabolomics has shown a consistent pattern of amino acid dysregulation across wasting conditions, likely related to the common development of resistance to anabolic stimuli and insulin. It has also shown major phospholipid classes are affected in sarcopenia and cachexia, providing a consistent lipid signature associated with muscle loss, but the involvement of phospholipids in disuse is less clear especially when an energy balance is maintained. If investigated further, this may provide a distinction between forms of muscle loss. Similarly, acylcarnitines are elevated in sarcopenia and following inactivity, likely relating to mitochondrial dysfunction in skeletal muscle, but are seemingly not affected by cachexia. However, mitochondrial energetics are clearly impacted in all forms of deconditioning suggesting a distinct aetiology for the dysregulation of mitochondrial function in cachexia. Likewise, metabolomics evidence suggests neuromuscular junction instability drives sarcopenia, but neuromuscular junctions are stable in cachexia. Future metabolomics research may be able to identify distinct aetiologies between conditions that will enable the development of targeted therapies. An overview of the major findings from untargeted metabolomics so far is provided in Figure 1.

However, there are several limitations to the current literature that should be addressed in future research. Although evidence suggests wider metabolic disturbances occur in wasting, there is very little focus in this area, allowing potential key insights into the aetiology of wasting to go undetected. The cross-sectional design of many metabolomics studies means causal links between metabolite level and disease cannot be established and prevents metabolic changes being assessed over time. We therefore cannot identify where in the metabolic pathway dysregulation occurs, preventing effective interventions from being developed. Additionally, differences in operational definitions of sarcopenia and cachexia have led to some discrepancies. Diagnostic criteria should be carefully considered in study design to ensure full clinical relevance of the results. Finally, targeted metabolomics is required for full validation of metabolite identification and biomarker discovery, however, when compared to untargeted analysis there are few targeted studies currently available. This is a key limitation as it means the validation stage of the metabolomics workflow is missing and prohibits the clinical translation of results.

## Abbreviations

BCAA: Branched chain amino acid; CSA, cross-sectional area; EAA, essential amino acid; HOMA-IR, homeostatic model assessment of insulin resistance; HSL, hormone sensitive lipase; MPB, muscle protein breakdown; MPS, muscle protein synthesis; TCA, tricarboxylic acid
